# The transcutaneous electromyography recording method for intraoperative neuromonitoring of recurrent laryngeal nerve during minimally invasive parathyroidectomy

**DOI:** 10.1038/s41598-020-64675-0

**Published:** 2020-05-06

**Authors:** Peng Li, Qing-Zhuang Liang, Dong-Lai Wang, Bin Han, Xin Yi, Wei Wei, Feng-Yu Chiang

**Affiliations:** 1 Department of thyroid and parathyroid Surgery, Peking University Shenzhen hospital, Peking University Health Science Center, Shenzhen, China; 2 Department of Otorhinolaryngology, E-Da Hospital, I-Shou University, Kaohsiung, Taiwan

**Keywords:** Action potential generation, Action potential generation, Parathyroid diseases, Parathyroid diseases

## Abstract

Intraoperative neuromonitoring (IONM) facilitates recurrent laryngeal nerve (RLN) protection in thyroid and parathyroid surgeries. This study aimed to investigate a novel transcutaneous electromyography (EMG) recording method for IONM of the RLN during minimally invasive parathyroidectomy (MIP). Twenty patients with primary hyperparathyroidism undergoing MIP were enrolled. Two paired needle electrodes were percutaneously inserted into the bilateral laminas of thyroid cartilage for monitoring the vagus nerve and RLN. A standardized IONM procedure (V_1_-R_1_-R_2_-V_2_ signals) was strictly followed, and the RLN was routinely located and mapped. Pre- and postoperative laryngofiberoscopy was performed to confirm vocal cord function. The proposed technique was successfully used in all patients, and typical EMG signals were effectively detected. No significant change in EMG signals before and after tumor resection was noted, and a normal vocal cord movement was ensured in all patients with postoperative laryngofiberoscopy. IONM helped localize the position of the RLN and facilitated the safe resection of the parathyroid tumor during MIP. The novel transcutaneous EMG recording method proposed in this study was feasible, convenient, reliable, and inexpensive.

## Introduction

Recurrent laryngeal nerve (RLN) injury is one of the most common complications in thyroid surgery. Unilateral RLN injury can result in hoarseness, exerting a great impact on singers, teachers, and other professional voice users. Bilateral RLN injuries can lead to airway compromise, and emergent tracheostomy may be required. Furthermore, it has been reported as one of the most common causes of medicolegal litigation^1^. In recent decades, intraoperative neuromonitoring (IONM) has been increasingly applied in thyroid surgery; it is effective in RLN protection^2^. The application of IONM has seldom been reported in parathyroid surgery, although it has been considered to be equally important as thyroid surgery^3^.

The primary hyperparathyroidism (PHPT) can be caused by adenoma (85%), hyperplasia (15%), and carcinoma (1%–2%). The hypersecretion of parathyroid hormone (PTH) leads to persistent hypercalcemia and is clinically manifested as weakness in limbs, bone pain, recurrent urinary calculi, dry mouth, dyspepsia, and constipation^4^. Surgery is a favorable regimen for treating PHPT. Also, minimally invasive parathyroidectomy (MIP) has become highly popular among patients with a single parathyroid adenoma because of its minimal invasion and appropriate cosmetic features^5^. Clear identification of the RLN may be difficult in a narrow surgical field during MIP, which is potentially associated with a high risk of RLN injury due to the close anatomical relationship between the enlarged parathyroid and RLN. Therefore, locating and mapping the RLN are of great significance before tumor resection using IONM during MIP^6^.

Nowadays, the endotracheal tube–based nerve monitoring method (e.g., EMG tube) is widely accepted as a standard for IONM of the RLN. However, several limitations have been reported during the clinical use of EMG tubes^7–9^. Chiang first compared two different EMG recording methods between an EMG tube and thyroid cartilage (TC) electrodes and concluded that TC electrodes obtained higher and more stable EMG signals and fewer false results during the IONM of the RLN^10^. However, the trans-thyroid-cartilage neuromonitoring method becomes difficult when only a small skin incision is needed for surgery. This study aimed to investigate a novel transcutaneous EMG recording method for the IONM of the RLN during MIP.

## Results

A total of 20 patients successfully underwent MIP, including 9 men and 11 women, with a mean age of 48 years (range, 31–66 years). The preoperative serum level of calcium, phosphorus, and PTH was 10.80 ± 0.68 mg/dL, 2.67 ± 0.59 mg/dL, and 172.66 ± 118.44 pg/mL, respectively. On the next day after the surgery, the serum level of calcium, phosphorus, and PTH was 8.88 ± 0.48 mg/dL, 3.04 ± 0.71 mg/dL, and 13.96 ± 13.39 pg/mL, respectively. A significant difference in the serum levels of calcium and PTH was noted before and after the surgery (*P* < 0.0001), but no significant difference was noted in the serum phosphorus level (*P* = 0.0860). A total of 20 parathyroid tumors (8 superior parathyroids and 12 inferior parathyroids) were resected, and all were diagnosed as parathyroid adenomas by the pathological examination.

**Figure 1:**
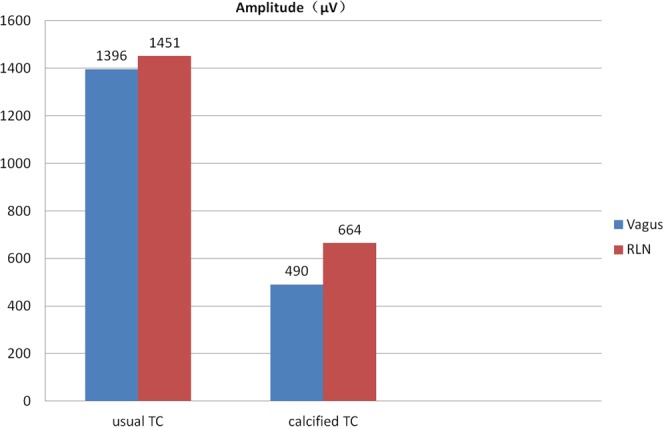
Mean EMG amplitudes elicited from vagus nerve and RLN were relatively lower in 6 patients with a calcified TC compared with those in 14 patients with a normal TC.

**Figure 2:**
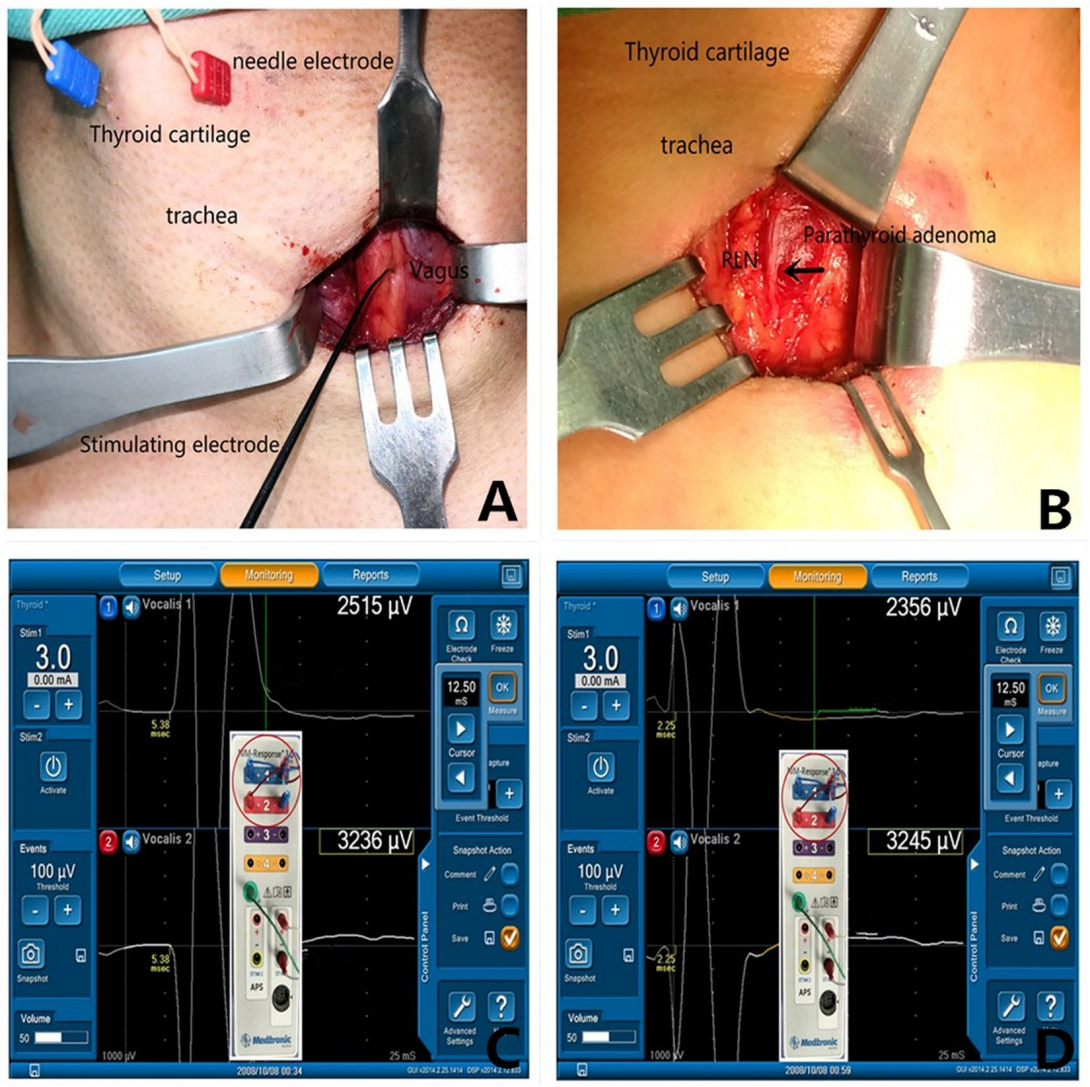
(**A**) Two paired needle electrodes were vertically inserted into the left and right TC laminas, and a 2-cm transverse incision was made on the natural skin crease. Vagus nerve stimulation was performed after opening the carotid sheath. (**B**) RLN (→) was found adhered to the surface of the left superior parathyroid adenoma. (**C**) Typical EMG waveform of vagus nerve stimulation with a latency of 5.38 ms after using the double channel method (red circle). (D) Typical EMG waveform of RLN stimulation with a latency of 2.25 ms after using the double channel method (red circle).

In all patients, the proposed IONM technique was successfully conducted, and typical EMG signals were effectively detected and recorded (Table 1). The mean EMG amplitude was 1124 ± 797, 1208 ± 741, 1350 ± 959, and 1272 ± 921 μV for V_1_-R_1_-R_2_-V_2_ signals, respectively. No significant change in the EMG amplitudes of the vagus nerve and RLN was noted before and after the parathyroid resection (V_1_ vs V_2_, *P* = 0.5896 and R_1_ vs R_2_, *P* = 0.6038). No significant change in the VHI-10 score was observed before and after the surgery (2.70 ± 4.35 vs 2.30 ± 3.80, *P* = 0.7585). In the process of needle electrode placement, TC electrodes were inserted easily in 14 patients. The needle electrodes were inserted with great difficulty in six patients owing to the calcified TC, and the initial EMG amplitudes were less than 500 μV (Fig. 1). Among eight patients with superior parathyroid tumors, the RLN was exposed and dissected in five patients due to its adhesion to the tumor (Fig. 2). In the other 12 patients with inferior parathyroid tumors, the RLNs were not actively exposed because the nerves had a relative distance from the tumors after mapping the exact nerve position (Table 1).

**Table 1:** Detailed information and recorded EMG signals for 20 patients.

No. hardness	Sex Age/ year Tumor location			TC	Amplitude (μV)			
					V_1_	R_1_	R_2_	V_2_
1	M	63	Inferior	U	463	549	474	478
2	M	44	Inferior	U	520	715	692	532
3	F	52	Superior	U	1480	1487	876	1808
4	M	42	Superior	U	620	680	651	550
5	F	62	Inferior	U	1364	1566	1520	1359
6	M	63	Inferior	U	560	774	650	644
7	F	46	Inferior	U	2400	963	2344	2862
8	F	32	Inferior	U	2395	1919	1912	2295
9	M	33	Inferior	U	1265	1405	1358	1214
10	F	33	Superior	U	3236	3245	2404	2489
11	F	55	Superior	U	1516	2098	2058	1452
12	F	31	Inferior	C	425	445	463	447
13	M	45	Superior	U	1087	1098	1420	1068
14	F	66	Inferior	C	622	832	994	656
15	F	50	Inferior	C	253	263	251	257
16	F	62	Inferior	C	451	519	493	467
17	M	34	Inferior	U	1210	1877	3588	3486
18	M	50	Superior	C	444	515	502	485
19	M	41	Superior	U	1429	1932	2026	1831
20	F	55	Superior	C	744	1290	1335	1070

C, Calcified TC; F, female; M, male; TC, thyroid cartilage; U, usual TC.

The postoperative laryngofiberoscopic examination confirmed the normal movement of vocal cords in all patients, and no one complained of voice change. No other complications, such as breaking of the needle electrode, subcutaneous or laryngeal hematoma, wound infection, and so forth, occurred. All patients were satisfied with the cosmetic outcomes of the wounds. After 6 months of follow-up, the serum calcium and PTH levels returned to the normal range in all patients.

## Discussion

At present, IONM has become an important adjunct for RLN protection in thyroid surgery, but it is underreported in parathyroid surgery^3^. In 2009, Kandil reported the necessity of IONM in minimally invasive video-assisted parathyroidectomy^11^ because the RLN was closely associated with the parathyroid tumor and at risk of injury in parathyroidectomy. The rate of RLN injury was reported to be 3.1% or 3.06% in thyroid and parathyroid surgeries^12,13^. The RLN is an important anatomical landmark for finding parathyroid tumors^3^. In most patients, the superior parathyroid tumors were located in the posterior region of the RLN, while the inferior parathyroid tumors were located in the anterior region of the RLN (Fig. 2). The use of IONM technology helped locate and map the RLN quickly, enabling the detection of tumors and protection of the nerves. In this study, the position of the RLN was routinely mapped before dissecting the posterior thyroid gland and resecting the parathyroid tumor. The RLN was not routinely exposed during MIP in this study, but it was exposed and dissected out if the nerve was found closer or adhered to the parathyroid tumor. The superior parathyroid tumor was often found to be associated with the RLN anatomically and had a higher risk of injury during tumor resection. Among eight patients with superior parathyroid tumors, the RLN adhered to the tumor in five nerves and needed to be exposed and dissected carefully to prevent nerve injury. However, in the other 12 patients with inferior parathyroid tumors, the RLN was not required to be exposed after mapping the nerve position and ensuring that the nerves were at a relative distance from the tumors. Therefore, the wound hemostasis was performed confidently without worrying about clamping or thermal injury of the RLN because the exact nerve position was known.

Previous studies reported the use of various EMG recording methods during the IONM of RLN, including intramuscular vocal cord electrodes placed endoscopically or through the cricothyroid membrane^14,15^ and laryngeal surface electrodes placed in the post-cricoid area or attached to an endotracheal tube^16,17^. In recent years, the endotracheal tube–based surface electrodes or EMG tubes have gained popularity because of the essential advantages of their easy setup and use, noninvasive nature, and capacity to derive larger areas of the target muscle. However, several limitations were encountered during the clinical use of the EMG tube, including the following: (1) verifying proper electrode–cord position and readjusting EMG tube is troublesome and time-consuming for anesthesiologists; (2) displacement of the EMG tube during surgical maneuvers on the thyroid lobe or trachea often occurs and may cause unstable EMG signals; (3) use of the EMG tube is difficult where IONM is unexpectedly required during the surgery; and (4) cost of the EMG tube is relatively high.

A variety of EMG signal recording methods to overcome the limitations of the EMG tube during the IONM of the RLN have been reported^10,18–29^ (Table 2). These methods are divided into three categories according to the way of electrode placement.

1. Needle electrode recording method:^10,19–22,25,27^ The needle electrodes are inserted into laryngeal muscles or tissues to record EMG signals. This method has the advantages of strong EMG signals, good stability, and convenient implantation. However, the major shortcoming was invasiveness.
2. Surface electrode recording method:^18,23,24,26^ The electrodes are brought into contact with the surface of laryngeal muscles or tissues to record strong and noninvasive EMG signals. However, stability is relatively poor. The displacement of the electrode or the liquid on the contact surface may influence the quality of IONM.
3. Adhesive skin-electrode recording method^28,29^. It is used to record EMG signals on the surface of the anterior larynx using adhesive electrodes. It is noninvasive, relatively stable, and convenient to operate, but low EMG signals limit the clinical application.

**Table 2:** Various EMG recording methods for IONM during thyroidectomy and parathyroidectomy.

Authors/year	Objects	Control group	Electrode type	Conclusions
Marcus B *et al*.^18^	Humans	No	Postcricoid surface electrode	The postcricoid surface electrode method was safe, simple, and effective for IONM
Petro ML *et al*.^19^	Humans	No	Transcricothyroid needle electrodes	This technique was sensitive, easy to use, and accurate
Haerle S *et al*.^20^	Humans	No	Postcricoid needle electrodes	This method was feasible and reliable compared to the standard vocal cord monitoring
Alon EE *et al*.^21^	Humans	No	Transcricothyroid needle electrodes	This method was a safe and reliable technique for bilateral monitoring of RLN
Chiang FY *et al*.^10^	Humans	EMG tube	TC needle electrodes	TC electrodes obtained higher and more stable EMG signals as well as fewer false EMG results
Farizon B *et al*.^22^	Humans	No	Transcricothyroid needle electrodes	This method predicted a postoperative vocal fold function during a bilateral thyroid surgery
Wu CW *et al*.^23^	Porcine model	EMG tube	TC surface electrode	This method confirmed the feasibility for recording EMG signals during IONM
Liddy W *et al*.^24^	Humans	EMG tube	TC surface electrodes	This method provided similar and stable EMG signals with equal sensitivity
Zhao Y *et al*.^25^	Porcine model	EMG tube	TC needle electrodes	EMG amplitudes were higher and identified RLN injury earlier than ET electrodes
Wu CW *et al*.^26^	Porcine model	EMG tube	Transcutaneous surface electrodes	This method was feasible, but amplitudes signals were lower
Li P *et al*.^27^	Humans	No	Arytenoid muscle needle electrodes	This method was considered to be safe, feasible, and reliable
Sung ES *et al*.^28^	Porcine model	EMG tube	Surface pressure sensor	These sensors were able to identify laryngeal twitching. The stimulus intensity was equivalent to that from conventional vocalis EMG
Lee HS *et al*.^29^	Humans	EMG tube	Adhesive skin electrodes	This method was successful in all nerves, but amplitudes signals were lower

In summary, it is believed that the ideal IONM method should have the following characteristics: strong EMG signals, noninvasiveness, stability, and convenience. However, no method fulfilling the aforementioned conditions is available to date. The endotracheal tube–based surface electrodes is still the suboptimal choice during thyroidectomy and parathyroidectomy.

Several clinical and animal studies have reported an alternative trans-thyroid-cartilage EMG recording method as feasible, stable, and convenient^10,26^. However, this recording method requires the exposure of the TC, limiting its use in procedures involving only a small neck skin incision, such as minimally invasive thyroidectomy (MIT) and MIP. The present clinical study reported a transcutaneous EMG recording method for the IONM of the RLN during MIP and verified its feasibility preliminarily. It was speculated that this method was worth further clinical research in MIT.

The EMG amplitudes in six patients with TC calcification were less than 500 μV (490 μV). Besides the learning curve, other possible reasons were also speculated:

1. When the RLN is stimulated, the innervated internal laryngeal muscle (except the cricothyroid muscle) can be induced to generate depolarization. Meanwhile, the EMG signal can diffuse into the skin through the TC and decline rapidly. For patients with TC calcification, the tip of the needle electrode is hardly inserted into the periosteum, which may be just under the skin.
2. The RLN should be dry, as the fluid or blood on the tissue can weaken the EMG signal, which could explain why the EMG amplitude of RLN in patient 3 decreased by 40% (from 1487 μV to 876 μV), while the EMG amplitude of VN increased from 1480 μV to 1808 μV.
3. TC calcification was evaluated in this study by evaluating the resistance of the insertion of the needle electrode. In some cases with high resistance, the needle electrode might not reach the periosteum to avoid the injury on the TC. Hence, it is better to use computed tomography (CT) to evaluate TC calcification.

In all patients, the IONM setup was completed within a few minutes, and no complication of subcutaneous or laryngeal hematoma caused by percutaneous insertion of recording electrodes into the TC occurred. In addition, the cost of using IONM technology is also an issue worth consideration. The cost of using an EMG tube in the hospital under study is about $750 each time, and the routine use is restricted by the medical insurance policy. However, the cost of using needle electrodes is only $100, which most patients can afford.

The novel transcutaneous EMG recording method proposed in this study showed reliable, feasible, and cheap. However, it still had some limitations:

1. The recording needles were only 12 mm in length and too short to be inserted into the TC in patients with an obese neck. However, EMG signals were still well recorded.
2. Inserting needle electrodes into a severely calcified TC could be difficult. Also, relatively lower EMG amplitudes (less than 500 µV) were noted in four of six patients with a severely calcified TC.
3. It was an invasive method, despite no complications, such as hemorrhage and TC injury in the present study. Further studies with a large number of cases should be conducted to verify the secure use of the proposed method.
4. The sample size was relatively small, and no control group was used in this study. Hence, further clinical studies are needed to verify the findings.

## Conclusions

IONM helps localize the position of the RLN and facilitate safe resection of parathyroid tumors during MIP. It also increases surgeons’ confidence and comfort through ensuring the functional integrity of RLN after parathyroid resection. The novel transcutaneous EMG recording method proposed in this study is a simple, reliable, and inexpensive way for recording the IONM of the RLN during MIP.

## Material and Methods

This study was conceived as a pilot study. Participants were recruited from the Department of Thyroid and Parathyroid Surgery, Peking University Shenzhen Hospital. The study was approved by the ethics committee of the Peking University Shenzhen Hospital (2019-025) and conducted according to the guidelines of the Declaration of Helsinki on biomedical research involving human participants. All participants provided written informed consent.

### Inclusion criteria of patients

From May 2016 to December 2017, patients with PHPT admitted to the Department of Thyroid and Parathyroid Surgery (Peking University Shenzhen Hospital, Shenzhen, China) were enrolled in this study. The inclusion criterion was as follows: a single parathyroid adenoma on the unilateral neck after imageological examinations (color Doppler ultrasound, CT, or single-photon emission CT). The exclusion criteria were as follows: multiple parathyroid adenomas, multiple endocrine adenoma type 1, suspected parathyroid carcinoma, tumors located in the thoracic cavity, need to undergo thyroid surgery simultaneously, and preoperative vocal cord palsy.

### Voice and laryngoscopic assessment

The electronic fiber laryngoscope and Voice Handicap Index 10 (VHI-10) scores were evaluated preoperatively. The electronic fiber laryngoscope was rechecked on the first day after the surgery. If the paralysis of vocal cords occurred, the monitoring was performed in months 1, 3, and 6 after the surgery, and it was considered as permanent vocal cord paralysis not recovered in 6 months. The VHI-10 score was rechecked on the third day after the surgery. All the aforementioned data were recorded in detail.

### IONM setup and surgical procedure

All patients underwent general anesthesia induced with lidocaine (1 mg/kg), propofol (4.0 μg/L), sufentanil (0.5 μg/kg), rocuronium bromide (0.3 mg/kg), and target-controlled infusion (TCI). After performing assisted ventilation for 5 min, regular endotracheal tubes were inserted assisted with a visible laryngoscope. The anesthesia was maintained as follows: propofol (3.5–4.0 μg/L) for TCI continuous intravenous pumping and remifentanil [0.1 μg/(kg · min)] for continuous intravenous pumping.

After successful anesthetic intubation, the shoulders were cushioned with pillows to achieve cervical hyperextension. Palpation was performed to determine the outline of the TC. Then, two paired needle electrodes (length, 12.0 mm; diameter, 0.4 mm; Medtronic Xomed Inc., FL, USA) were obliquely inserted about 1 cm from the midline of the thyroid cartilage into the left and right TC laminas (Fig. 2). The electrode wires were then fixed on a surgical drape with sutures. The channel leads from the needle electrodes were connected to the NIM-Response 3.0 (Medtronic Xomed Inc., FL, USA). On the patient interface, a “double channel” method (exchanging one electrode of channel 1 and channel 2) was adopted to amplify the EMG signal (Fig. 2). Channel 1 and channel 2 both represented the potential difference between the left and right sides, with little difference between them. To unify the standards, the data from the channel with higher EMG amplitude were chosen and registered. The monitoring system generated stimuli with a time window set to 50 ms and an amplitude scale set to 0.2 mV/division. The pulsed stimuli were 100 μs in duration and 4 Hz in frequency. The event capture was activated at a threshold of 100 μV, and the stimulus current was set at 3.0 mA.

Based on the position of the parathyroid tumor detected by preoperative localization, a 2-cm transverse incision was made on the natural skin crease. The space between the sternocleidomastoid muscle and the sternohyoid muscle was dissected to enter the carotid sheath, and then the internal jugular vein and common carotid artery were exposed. A handheld monopolar probe (ball tip, 1 mm; Medtronic Xomed Inc.) was used to stimulate the vagus nerve surface (without dissecting the carotid sheath), and the V_1_ signal was obtained (Fig. 2). A “cross method” was used to map the RLN. A monopolar probe was placed on the inferior pole of the thyroid to locate the RLN, providing the R_1_ signal. Once the R_1_ signal was obtained, the RLN was mapped upward along the tracheoesophageal groove from the location point. The RLN was not routinely exposed in this study, but it was exposed and dissected out if the nerve was found closer or adhered to the parathyroid tumor (Fig. 2).

After resecting the parathyroid tumor and submitting it for the frozen-section examination, the blood was drawn subsequently from the same side of the internal jugular vein after 10 min of tumor resection for intraoperative parathyroid hormone (IOPTH) analysis. If the IOPTH level decreased by more than 50% compared with that obtained preoperatively, the surgery was ended. After the complete hemostasis of the wound, the RLN function was routinely reconfirmed by stimulating the RLN and the vagus nerve. The R_2_ and V_2_ signals were obtained. Then, subcutaneous tissues and skin were sutured without placing a drainage tube.

### Data recording and processing methods

Microsoft Office Excel 2007 was used for recording data related to the sex and age of patients, position of the parathyroid tumor, hardness of the TC, laryngofiberoscopic examination, and pathological results. Moreover, the serum calcium, phosphorus, and PTH levels, amplitude, and latency of EMG before and after resection were expressed as mean ± standard deviation. GraphPad Prism 7.0 software was used to compare differences among all parameters before and after the surgery. A *P* value ≤ 0.05 was considered statistically significant.

## Supplementary information

Supplementary information.
