# Supplementary material for: The transcutaneous electromyography recording method for intraoperative neuromonitoring of recurrent laryngeal nerve during minimally invasive parathyroidectomy
Source: Sci Rep. 2020 May 6;10:7609. doi: 10.1038/s41598-020-64675-0 (PMC7203296; doi:10.1038/s41598-020-64675-0)
Supplement: Supplementary file 1 — Supplementary information. [file 41598_2020_64675_MOESM1_ESM.pdf]

The transcutaneous electromyography recording method for intraoperative neuromonitoring of recurrent laryngeal nerve during minimally invasive parathyroidectomy.

Authors:

Peng Li[1](ORCID 0000-0002-1150-5915), Qing-Zhuang Liang[1], Dong-Lai Wang[1], Bin Han[1],Xin Yi[1],Wei Wei[1], Feng-Yu Chiang*[2]

[1]Department of thyroid and parathyroid Surgery, Peking University Shenzhen hospital, Peking University Health Science Center, Shenzhen, China.

[2] Department of Otorhinolaryngology –E-Da Hospital, Kaohsiung, Taiwan.

Send correspondence to Feng-Yu Chiang,MD, Department of Otolaryngology, E-Da Hospital, No.1, Yida Road, Yanchao District, Kaohsiung, 82445, Taiwan

E-mail: fychiang@kmu.edu.tw

NO.	VHI-10(Preoperative)	VHI-10(Postoperative)
1	0	0
2	0	0
3	2	0
4	0	0
5	17	14
6	0	0
7	0	0
8	0	0
9	0	4
10	6	4
11	0	0
12	6	0
13	0	0
14	10	10
15	0	0
16	5	5
17	0	0
18	3	4
19	0	0
20	5	5
